# Immune Thrombocytopenia in a 94-Year-Old Woman: Challenges in Diagnosis and Management of an Uncommon Presentation in the Elderly

**DOI:** 10.7759/cureus.86417

**Published:** 2025-06-20

**Authors:** Katherine Thornburgh, Taylor F Faust, Thomas L Horton

**Affiliations:** 1 Department of Research, Alabama College of Osteopathic Medicine, Dothan, USA; 2 Department of Family Medicine, Jackson Hospital, Montgomery, USA

**Keywords:** elderly falls, geriatrics, hematology, immune thrombocytopenia (itp), immunology

## Abstract

We describe a unique case of a 94-year-old woman who presented to the emergency department with a large hematoma on her left lateral thigh following a fall. Laboratory workup revealed a platelet count of 10,000/μL, and incidentally, just a week prior to her presentation in the emergency department, her platelet count was 26,000/μL. After consultation with hematology/oncology, a diagnosis of immune thrombocytopenia (ITP) was established, and she was given 40 mg dexamethasone, transfused with one unit of platelets, and initiated on intravenous immunoglobulin (IVIG). She returned to the emergency department a week later due to severe fatigue and transient loss of consciousness following IVIG administration. This case highlights the complexities of diagnosing and managing ITP in elderly patients, emphasizing the need for individualized treatment strategies to balance efficacy and safety.

## Introduction

Immune thrombocytopenia (ITP) is a rare autoimmune disorder where the immune system mistakenly targets and destroys platelets, which are essential components of normal blood clotting [1]. The destruction of platelets leads to a low platelet count, increasing the risk of bruising, bleeding, and related complications such as petechiae, purpura, heavy menstrual periods, and slower wound healing [2]. ITP can present as either an acute or chronic event. Acute ITP, the most common form, lasts less than six months and primarily affects children of both sexes. In contrast, chronic ITP persists for more than six months, predominantly affecting adults, especially women, who are affected two to three times more often than men [3]. ITP is most commonly diagnosed in women aged 15 to 59, with symptoms most frequently appearing in those in their 30s and 40s [3]. The annual incidence of ITP in adults ranges from one to six cases per 100,000 [4]. The prevalence of chronic disease in adults is approximately 12 per 100,000 cases [3]. 

ITP symptoms can range from being entirely asymptomatic to severe bleeding that is difficult to control and may occur internally, under the skin, or from the skin itself. Bleeding can be categorized into: petechiae (small, flat red spots under the skin caused by blood vessel leakage), purpura (red, purple, or brownish skin discoloration due to bleeding), and hematomas (collections of clotted or partially clotted blood under the skin). Other symptoms may include nosebleeds, blood in the urine or stool, heavy menstrual bleeding, and extreme fatigue [5]. 

The diagnosis of ITP involves a thorough medical and clinical history. Blood tests can provide some insights, such as a complete blood count (CBC), which measures the platelet count and the number of other blood cells. A blood smear provides a microscopic view of platelets, while bone marrow tests, including aspiration, help rule out other platelet disorders [1]. Additionally, antibody tests may be conducted for patients at risk for HIV, hepatitis C, or *Helicobacter pylori*, as these conditions are comorbid with ITP. ITP can thus be viewed as an acute or chronic event. 

Infection and immune changes are among the most common events that can trigger ITP, with viral illnesses often preceding infection-associated ITP. This occurs because antibodies are produced to fight viral antigens, leading to cross-reactions with normal platelet antigens due to molecular mimicry [1]. Common viral infections associated with ITP include: HIV, hepatitis C, cytomegalovirus, and varicella zoster [1]. ITP can also be associated with autoimmune alterations due to the development of autoantibodies. Conditions like antiphospholipid syndrome, systemic lupus erythematosus (SLE), Evans syndrome, and others can trigger autoimmune-mediated ITP [6]. Various medications, such as abciximab, gold compounds, heparin, linezolid, as well as malignancies like chronic lymphocytic leukemia and adenocarcinoma, and some endocrine disorders like hypothyroidism and Addison's disease, can lead to ITP [7]. ITP can arise through various mechanisms, making it essential to conduct a thorough and diligent evaluation to accurately diagnose and identify potential underlying causes.

This report explores the case of a 94-year-old woman whose presentation added a layer of complexity to her medical course. Her hospital stay and the management of her ITP diagnosis are discussed in detail. 

## Case presentation

A 94-year-old woman presented to the emergency department after a ground-level fall onto her left hip. She reported generalized weakness but denied dizziness, head trauma, or loss of consciousness at the time of the fall. 

The patient's past medical history included a record of flank pain, gastroesophageal reflux disease (GERD), renal angle tenderness, hypertensive disorder, enteritis, and suspected cardiovascular disease. Her surgical history encompassed hysterectomy, cataract repair, bilateral hip replacements, right knee replacements, and tonsillectomy. She was a nonsmoker and denied the use of alcohol or illicit drugs. Significant family history included her father passing away from myocardial infarction at age 65, her mother having a history of heart disease, and her sister being diagnosed with brain cancer of an unknown type. The patient's medication list included amlodipine, docusate sodium, eltrombopag, losartan, metoclopramide, ondansetron, pantoprazole, polyethylene glycol 3350, prochlorperazine, and rosuvastatin.

Physical examination revealed a 10x5-inch hematoma on the left lateral thigh, with no signs of active bleeding or neurological deficits. During her stay in the emergency department, she underwent radiographic imaging, which ruled out any fractures. An abdominal CT performed at the time of emergency department visit showed no findings reported by the radiologist for the spleen or liver. A prior CT of the abdomen/pelvis was completed in 2012, which was reported as homogeneous and normal. 

**Figure 1:**
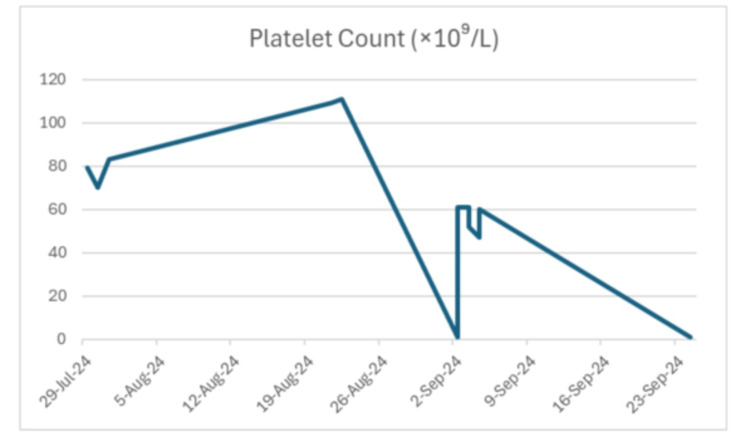
Platelet count recorded during the patient's multiple hospitalizations

Laboratory workup (Table 1) indicated a platelet count of 10,000/μL, a significant decline from 26,000/μL just seven days prior, and a decrease of more than 120,000/μL from the normal range. Platelet counts by date, including the patient's baseline and values recorded during multiple hospitalizations, are summarized in Figure 1. A peripheral blood smear (PBS) showed isolated thrombocytopenia without platelet clumping, schistocytes, or other abnormalities. Coagulation studies, along with liver and renal function tests, were within normal limits.

**Table 1:** Initial blood work and follow-up emergency department visit blood work seven days later CBC: Complete blood count; WBC: White blood cell; RBC: Red blood cell; MCV: Mean corpuscular volume; MCH: Mean corpuscular hemoglobin; MCHC: Mean corpuscular hemoglobin concentration; RDW: Red cell distribution width; MPV: Mean platelet volume; NRBC: Nucleated red blood cell; Abs: Absolute

Test	Result	Reference Range
CBC automated with differential - initial workup		
WBC count	5.6 k/cumm	3.1-9.5 k/cumm
RBC count	3.93 m/cumm	3.90-5.21 m/cumm
Hemoglobin	11.7 g/dL	11.9-15.3 g/dL
Hematocrit	36.50%	35.9-46.8%
MCV	92.9 fL	82.6-96.2 fL
MCH	29.8 pg	27.0-32.3 pg
MCHC	32.1 g/dL	31.2-35.1 g/dL
RDW	13.60%	11.4-14.0%
Platelet count	79 k/cumm	140-440 k/cumm
MPV	10.4 fL	9.0-11.8 fL
Differential type	Manual	
Segmented	68%	40.0-89.0%
Lymphocytes	21%	15.0-50.0%
Monocytes	6%	0.0-14.0%
Eosinophils	5%	0.0-7.0%
Basophils	0%	0.0-2.0%
Immature granulocytes	1.3%	0.0-0.7%
NRBC	0	
Segmented (Abs)	3.8 k/cumm	1.7-6.1 k/cumm
Lymphocytes (Abs)	1.2 k/cumm	1.1-2.8 k/cumm
Monocytes (Abs)	0.3 k/cumm	0.3-0.9 k/cumm
Eosinophils (Abs)	0.3 k/cumm	0.0-0.3 k/cumm
Basophils (Abs)	0 k/cumm	0.0-0.1 k/cumm
Immature granulocytes (Abs)	0.1 k/cumm	0.0-0.1 k/cumm
Atypical lymphocytes	Few	
CBC automated with differential - seven days after initial workup		
WBC count	9.3 k/cumm	3.1-9.5 k/cumm
RBC count	4.28 m/cumm	3.90-5.21 m/cumm
Hemoglobin	12.6 g/dL	11.9-15.3 g/dL
Hematocrit	38.30%	35.9-46.8%
MCV	89.5 fL	82.6-96.2 fL
MCH	29.4 pg	27.0-32.3 pg
MCHC	32.9 g/dL	31.2-35.1 g/dL
RDW	13.50%	11.4-14.0%
Platelet count	109 k/cumm	151-374 k/cumm
MPV	10.8 fL	9.0-11.8 fL
Differential type	Auto	
Segmented	77.30%	43.3-72.4%
Lymphocytes	16.00%	16.0-44.3%
Monophils	5.70%	5.4-12.0%
Eosinophils	0.20%	0.1-4.3%
Basophils	0.20%	0.2-1.3%
Immature granulocytes	0.60%	0.0-0.7%
NRBC	0	
Segmented (Abs)	7.2 k/cumm	1.7-6.1 k/cumm
Lymphocytes (Abs)	1.5 k/cumm	1.1-2.8 k/cumm
Monocytes (Abs)	0.5 k/cumm	0.3-0.9 k/cumm
Eosinophils (Abs)	0 k/cumm	0.0-0.3 k/cumm
Basophils (Abs)	0 k/cumm	0.0-0.1 k/cumm
Immature granulocytes (Abs)	0.1 k/cumm	0.0-0.1 k/cumm

Given the acute decline in platelet count and absence of hematologic abnormalities, the hematology/oncology specialist was consulted for further evaluation. The patient was diagnosed with ITP and started on 40 mg of dexamethasone daily for four days. Due to her large hematoma and profound thrombocytopenia, she received a platelet transfusion. 

Despite initial improvement, she returned to the emergency department seven days later with severe fatigue and transient loss of consciousness, which occurred one day after receiving outpatient intravenous immunoglobulin (IVIG) treatment. While it cannot be definitively concluded that IVIG caused the patient’s fatigue and transient loss of consciousness, these symptoms, particularly fatigue, have been reported as potential side effects of IVIG therapy [8]. Repeat blood work showed an increase in platelet count to 109,000/μL. After stabilization and observation, she was discharged with close outpatient follow-up. 

## Discussion

ITP is an autoimmune disorder that causes immune-mediated platelet destruction, leading to thrombocytopenia and an increased risk of bleeding due to the blood’s inability to clot. This condition is usually a diagnosis of exclusion, characterized by isolated thrombocytopenia caused by IgG autoantibodies targeting circulating platelets [1]. Laboratory findings typical of ITP show platelet counts below 100,000/μL (reference range 150,000 to 400,000/μL), with normal white blood cell and hemoglobin levels [9]. 

ITP has two variants: primary, which is idiopathic, and secondary, often associated with drug reactions, autoimmune disorders like SLE, chronic lymphocytic leukemia, and infections such as HIV. The IgG autoantibodies target platelet membrane proteins GP IIb/IIIa, Ib/IIa, and VI in response to an inciting agent [1]. One mechanism contributing to thrombocytopenia is the accelerated clearance of platelets by macrophages, resulting in a shortened platelet half-life [2]. Another proposed mechanism involves T-cell-mediated cytotoxicity affecting bone marrow megakaryocytes, although further research is required to confirm this pathway [10]. 

Our patient's case is notable due to her advanced age (94 years old). ITP is most commonly diagnosed in women aged 30 to 40 years, making this presentation rare [6]. Elderly patients present unique challenges, including a higher risk of bleeding, increased treatment complications, and the necessity for individualized management strategies to mitigate potentially harmful treatment side effects [1]. 

ITP is identified by ruling out other causes of thrombocytopenia and conditions with similar symptoms and underlying mechanisms. The initial evaluation of ITP in both children and adults includes a comprehensive laboratory workup, starting with a CBC with differential, reticulocyte count, and PBS. Patients with ITP typically show normal white blood cell count, hemoglobin concentration, red cell indices, and leukocyte differential count, while the key indicator of ITP is a platelet count below 100,000/μL. For cases of moderate or severe thrombocytopenia, prothrombin time and activated partial thromboplastin time are also assessed, especially before planned invasive procedures or when bleeding is present. Patients undergoing splenectomy as a treatment for ITP may be screened for thyroid dysfunction beforehand due to the elevated risk of perioperative complications [1]. 

Management of ITP usually begins with corticosteroids, IVIG, or anti-D immune globulin for rapid platelet recovery in patients with Rh-positive blood type [7]. Given the timing of IVIG coinciding with our patient's return to the hospital due to fatigue and a transient loss of consciousness, it is vital to discuss several side effects to monitor in patients receiving this therapy. Side effects of IVIG can occur either immediately or in a delayed manner and may range from mild to severe. Mild reactions often include headache, low-grade fever, chills, and fatigue symptoms, as observed in our patient. Moderate side effects may involve vomiting, arthralgia, or chest pain, while severe adverse effects can include hypertension, anaphylaxis, bronchospasm, and altered consciousness. The episode of transient loss of consciousness experienced by our patient, which is considered a serious reaction, underscores the importance of closely monitoring elderly patients receiving IVIG, who may be more susceptible to complications and may have a harsher response to this treatment [5]. 

These agents are employed to rapidly raise platelet counts in emergency settings but are not suitable for long-term therapy due to their brief response duration and potential harmful and toxic effects [11]. For patients with persistent or chronic ITP who do not require immediate treatment, splenectomy and rituximab are commonly utilized alternative options [12]. Additionally, second- and third-line treatments may involve corticosteroids combined with immunosuppressive agents for patients unresponsive to monotherapy, offering a multi-target approach [13]. Splenectomy, a third-line option for those who have not responded to corticosteroids, is effective but limited by the risk of surgical complications, with no specific guidelines on timing for ITP patients [14]. It is an effective treatment but is constrained by the surgical risks and the absence of specific recommendations regarding the timing of splenectomy in ITP patients as part of current guidelines [12]. Other immunosuppressive agents, such as thrombopoietin receptor agonists (TPO-RAs) like eltrombopag and romiplostim, are used for patients unresponsive to corticosteroids, immunoglobulins, or splenectomy. These agents activate thrombopoietin (TPO) receptors on megakaryocytes, stimulating platelet production through the Janus kinase 2 (JAK2) protein tyrosine kinase and signal transducer and activator of transcription 5 (STAT5) pathways [15]. While these alternative treatments have been included in management guidelines, further research and clinical application are needed to optimize their use in ITP. 

If evaluated again, a more thorough approach would involve ruling out all potential causes of thrombocytopenia before confirming an ITP diagnosis. A more extensive workup could include assessing secondary causes of thrombocytopenia, such as liver disease, hypersplenism, bone marrow disorders, and infectious etiologies. In addition to routine blood tests, specific assays like platelet antibody tests or a bone marrow biopsy could help confirm the absence of other hematological conditions before diagnosing ITP. 

Diagnosing ITP remains challenging, as it is primarily a diagnosis of exclusion that requires clinicians to thoroughly rule out other causes of thrombocytopenia through a detailed history, physical examination, and extensive laboratory testing. Management becomes even more complex in elderly patients due to their increased risk of bleeding and a higher likelihood of adverse treatment reactions, as seen in our case with a suspected reaction to IVIG. This population also necessitates careful selection of therapies such as corticosteroids, immunoglobulins, and, when necessary, splenectomy. Given the variability in treatment response, limited long-term options, and the lack of standardized guidelines, especially for older adults, this case emphasizes the importance of individualized care and close monitoring.

## Conclusions

We present the case of a 94-year-old female patient with a history of hypertension, diagnosed with ITP following a significant drop in her platelet count after a fall. She initially responded well to treatment with corticosteroids and a platelet transfusion, which quickly improved her platelet count. Due to her advanced age and increased bleeding risk, this conservative approach was chosen to achieve a rapid response while minimizing potential side effects. IVIG was also used with caution, given its known adverse effects in older patients and its suspected association with the patient's subsequent fatigue and transient loss of consciousness. This case is particularly noteworthy, as ITP is rare in patients of this age group and requires individualized management due to age-related vulnerabilities, multiple comorbidities, and heightened sensitivity to possible treatment toxicity. Ongoing outpatient follow-up with hematology is essential for monitoring platelet stability, evaluating the potential for relapse, and determining the need for long-term therapy or escalation of care if necessary.

Ultimately, this case highlights the importance of tailoring ITP management strategies to the unique needs of elderly patients and underscores the value of careful treatment selection, adverse event monitoring, and proactive follow-up in this vulnerable population. It adds to the limited but growing body of literature on ITP in geriatric patients and supports the need for age-conscious clinical decision-making in both diagnosis and treatment.
